# Factors affecting the benefit of glasses alone in treating childhood amblyopia: an analysis of PEDIG data

**DOI:** 10.1186/s12886-023-03116-8

**Published:** 2023-09-28

**Authors:** Rosa Hernández-Andrés, María Josefa Luque, Miguel-Ángel Serrano, Andrew Scally, Brendan T Barrett

**Affiliations:** 1 Department of Optics and Optometry and Vision Science, University of Valencia, Doctor Moliner, 50, 46100 Burjassot, Spain; 2 Department of Psychobiology, University of Valencia, Avda. Blasco Ibañez, 13, 46010 Valencia, Spain; 3 School of Clinical Therapies, University College Cork, College Road, T12 K8AF Cork, Republic of Ireland; 4 School of Optometry & Vision Science, Phoenix South West Building, University of Bradford, BD7 1DP Bradford, West Yorkshire UK

**Keywords:** Amblyopia, Optical treatment, Visual acuity, Stereoacuity, Interocular difference

## Abstract

**Background:**

To evaluate factors associated with better outcomes from optical treatment alone in amblyopic children from 3 up to 7 years.

**Methods:**

Data extracted from two studies with similar protocols, Amblyopic Treatment Studies 5 (n = 152) and 13 (n = 128) from the Pediatric Eye Disease Investigator Group database, were used to determine by regression analysis the factors associated with improvements in visual acuity in the amblyopic eye, inter-ocular visual acuity difference and stereoacuity. Input variables were aetiology of amblyopia (anisometropic, strabismic and combined-mechanism amblyopia), treatment compliance, visual acuity, interocular visual acuity difference, stereoacuity, tropia size at distance and near, age and refractive error at baseline.

**Results:**

Despite the range of clinical factors considered, our models explain only a modest proportion of the variance in optical treatment outcomes. The better predictors of the degree of optical treatment success in amblyopic children are visual acuity of the amblyopic eye, interocular visual acuity difference, stereoacuity, treatment compliance and the amblyopic eye spherical-equivalent refractive error. While the aetiology of the amblyopia does not exert a major influence upon treatment outcome, combined-mechanism amblyopes experience the smallest improvement in visual acuity, tropia and stereoacuity and may need longer optical treatment periods.

**Conclusions:**

While results identify the factors influencing optical treatment outcome in amblyopic children, clinicians will be unable to predict accurately the benefits of optical treatment in individual patients. Whether this is because relevant clinical or non-clinical factors (e.g. nature and volume of daily activities undertaken) influences the outcomes from optical treatment has not yet been identified and remains to be discovered.

**Supplementary Information:**

The online version contains supplementary material available at 10.1186/s12886-023-03116-8.

## Key message

- The specific benefit of optical correction is already established and this has led to a change in amblyopia management whereby treatment starts with the provision of optical correction alone.
- The best predictors of the degree of optical treatment success in amblyopic children are visual acuity of the amblyopic eye, interocular visual acuity difference, stereoacuity, treatment compliance and the amblyopic eye spherical-equivalent refractive error.
- Overall, optical treatment alone resolved amblyopia in a low percentage of the amblyopic children. Furthermore, based on statistical modelling, clinicians will not be able to predict accurately the benefits of optical treatment for each individual patient.

## Introduction

Amblyopia is a unilateral, or infrequently bilateral, developmental disorder of vision associated with significant refractive error in one or both eyes and/or a misalignment of the visual axes [1]. It is usually associated with anisometropia (anisometropic amblyopia), strabismus (strabismic amblyopia) or their combination (combined mechanism) [1]. Its treatment typically consists of correcting the refractive error in combination with occlusion or optical penalization of the better eye, although alternatives to the latter methods (e.g. involving the use of dichoptic displays, virtual reality and perceptual learning) are the subject of considerable clinical research interest at present [2, 3].

During the past 20 or so years, the value of the optical correction component in amblyopia treatment has been recognized. In the past, it was common practice for patching or penalization to commence around the same time as the refractive correction was issued, making it impossible to distinguish the distinct therapeutic benefit of optical treatment from the benefits produced by, for example, occlusion. However, starting with Stewart et al. [4], the specific benefit of optical correction has been established and this has led to a change in amblyopia management whereby treatment starts with the provision of optical correction alone, typically for a minimum period of ~ 4 months [5] or longer if visual acuity continues to improve with glasses alone [6, 7]. The period of time when glasses represent the sole means of treatment can be labelled the “optical treatment alone” (OTA) period [8] and this is the subject of interest in the present study. The benefit of OTA in the treatment of amblyopia relates to the gains in visual acuity (VA) from wearing glasses for a sustained period of time beyond the gains which result from simple elimination of the optical blur when glasses are first worn. Thus, the benefit of OTA can be quantified by determining the improvement in VA in the affected eye from the time when refractive correction is first provided to the point when (typically after 18–20 weeks) the visual acuity ceases to improve with glasses alone [4, 5, 7].

What clinical factors affect the magnitude of the benefit from OTA? A number of studies have shown that OTA is beneficial regardless of the presumed etiology or severity of the amblyopic visual loss [8, 9]. A recent meta-analysis on the benefits of OTA in amblyopia [10] found that the optical treatment of amblyopia resulted in moderate-to-large effect sizes. Regression analysis of the data from the studies included in the meta-analysis found that the effect of optical treatment decreased with age and increased with treatment duration. Surprisingly, the analysis also found that better initial acuity was associated with larger effect sizes from OTA.

Two of the studies included in this meta-analysis were conducted by the Pediatric Eye Disease Investigator Group (PEDIG). These studies benefit from detailed protocol descriptions, large numbers of participants and an assessment of compliance with spectacle wear. These are the Amblyopia Treatment Studies (ATS) 5 & 13 and they examined the benefit of OTA in anisometropic amblyopia, in strabismic and in combined strabismic - anisometropic amblyopia [8, 9]. These PEDIG studies were conducted in children aged from 3 to < 7 years and they had virtually identical protocols in the OTA phase. In addition to examining the effect of age, presumed aetiology of the amblyopic visual deficit and the visual acuity at the onset of the OTA phase (VAAE, visual acuity of the amblyopic eye at baseline), the large participant numbers in the two studies also allows examination of whether additional factors were associated with better treatment outcomes. These additional clinical factors included the refractive error in the amblyopic eye, the magnitude of any anisometropia present, the magnitude of the angle of the strabismus (if present) and the level of stereoacuity at baseline. The results of the two studies show some differences from one another and from the conclusions of the meta-analysis referred to above. For example, as indicated, the meta-analysis found that bigger OTA effect sizes were found in eyes with better baseline acuity in the amblyopic eye. However, while the same finding emerged from the PEDIG study of anisometropic amblyopes (ATS5), the treatment effect sizes were not found to be associated with baseline amblyopic eye visual acuity in combined mechanism amblyopia, and to be associated with worse baseline amblyopic visual acuity in eyes with strabismic amblyopia when no anisometropia was present (ATS13). These results suggest that the aetiology of amblyopia may be important when it comes to the factors influencing treatment outcome. However, in the study by Stewart et al. [5] and in the recent meta-analysis [10], the effect size was not found to depend upon amblyopia etiology. Similarly, the impact of anisometropia magnitude upon treatment appears to differ between studies. In combined mechanism amblyopia, the improvement in VAAE was not associated with the magnitude of anisometropia [8] but in the study of anisometropic amblyopes, better treatment outcomes were found to be associated with lesser amounts of anisometropia [9].

By combining the results from two large PEDIG studies that have included essentially the same protocol in the OTA phase, our aim is to obtain a clearer picture of the factors that influence amblyopia treatment outcomes that arise from the optical treatment of amblyopia. We anticipate the results of this analysis will be useful to clinicians treating childhood amblyopia and to clinical researchers in identifying the factors that are, or appear to be, associated with better treatment outcomes from the optical treatment of amblyopia.

## Methods

The study has been evaluated by the “Ethics Committee” of University of Valencia (Spain) and deemed not to require ethics approval.

**Fig. 1:**
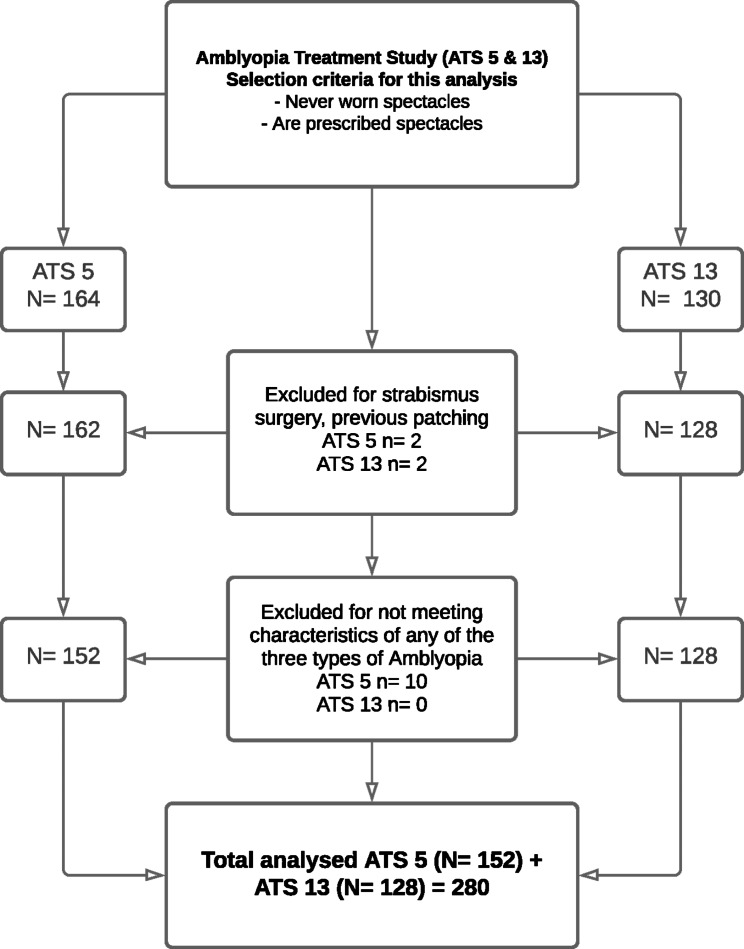
Selection criteria and participant numbers in the two Amblyopia Treatment Studies (ATS), ATS5 and ATS13. The studies are similar until the end of the OTA phase, and diverge thereafter. However, this analysis focuses on gains up to the end of the OTA phase and thus the results from ATS 5 and ATS 13 are combined. Total participants n = 280 [(n_ATS5_= 152) + (n_ATS13_= 128)]. Data sets for both studies are available on the PEDIG website [11]

Patients who had not previously worn glasses nor had any other form of treatment for amblyopia before enrolling in the PEDIG research studies [9] were selected from the ATS5 and ATS13 data sets, available on the PEDIG website [11]. In total, there were data available for 280 patients, (n_ATS5_=152, n_ATS13_=128 , see Fig. 1).

The methodological details for the studies are available elsewhere [9]. In brief, in both studies the VA was measured with ATS single-surround HOTV on the Electronic Visual Acuity tester [12–13], within a half-hour of when the newly-prescribed glasses were first worn. VA was monitored at subsequent at follow-up visits until it ceased to improve, ending the OTA period. Treatment compliance was classified in four categories: Excellent, Good, Fair and Poor [11] and was recorded by diaries. As these were multicentric trials, each child was monitored by a researcher at the centre where they enrolled in the trial. They were assessed by the same researcher at each centre. In cases where this compliance classification changed between visits, the worst level was used. Stereoacuity was measured with Titmus Fly and Randot Preschool Stereoacuity tests (Stereo Optical Co., Chicago, IL), both at the beginning and at the end of the OTA phase in study ATS13 but only at the end of the OTA phase in ATS5. This limits the extent to which this analysis can examine the change in stereoacuity that accrue from OTA, and the association between the stereoacuity at the beginning of the OTA phase and the improvements in VAAE arising from optical treatment.

In the ATS5 study, follow-up visits were conducted every 5 (± 1) weeks. Amblyopia was considered “resolved” in patients achieving an interocular acuity difference in VA of ≤ 1 line [9]. At this point, these patients continued using their spectacle correction but they did not have any other form of treatment. They had a final follow-up visit 4 to 6 months later [9]. In the ATS13 study, follow-up visits were only conducted every 9 weeks [8].

The PEDIG group follows very detailed protocols [11] for the refraction of participants, always taking the cycloplegic as a starting point. For the purposes of considering the possible impact of refractive error upon the magnitude of VA improvement during the period of OTA, the spectacle refractive error and not the cycloplegic refraction was used in our analyses. This is because the spectacle refraction is the refractive correction which is worn by the patient during the OTA. For analysis purposes, refraction has been converted to vectorial format [14] (M, J_0_, J_45_) and anisometropia was calculated as the difference between the spherical-equivalent (M) components of the right and left eye of each participant. There were no significant differences between the initial and final results for M, J_0_ and J_45_. Visual acuities in Snellen notation were converted to logMAR notation and the interocular difference in logMAR VA (IOD-VA) computed for each visit. Raw stereoacuity scores in ATS5 and ATS13, were converted into a categorical scale as follows: 1 = 40”; 2 = 60”; 3 = 100”; 4 = 200”; 5 = 400”; 6 = 800”. Stereoacuity thresholds worse than 800” were assigned to category 7. The classification of amblyopia types as anisometropic (A), strabismic (S) and combined-mechanism (C) followed the PEDIG criteria [8]. Relative to baseline, which is the moment when the new glasses were first worn, the benefits of OTA were assessed at the following time points: a) 6 to 10 weeks ± 3 days from baseline, b) 14 to 18 weeks ± 3 days from baseline and c) last visit when participants entered the next phase (i.e. beyond OTA) of ATS5 or ATS13. The total number of participants with data at each of these time points was 148 (n_ATS5_ = 70; n_ATS13_=78).

### Data analysis

SPSS version 26 and MATLAB were used for statistical analysis. Non-parametric statistics have been used because the variables do not follow a normal distribution. The clinical factors associated with better treatment outcomes were identified by partial correlation analysis and included in stepwise linear regression models with the Akaike [15] information criterion, to determine which clinical parameters influenced the OTA treatment outcome, measured as the final values of, and the changes in VAAE, IOD-VA and stereoacuity. Three categorical variables, amblyopia aetiology (anisometropic, strabismic and mixed), stereopsis level and strabismus type (none, distance, near or both) were considered. Continuous variables were age, distance and near tropia horizontal size, the three components of the refraction vector (M, J_0_, J_45_) and the VAAE at baseline. In all test p-values below 0.05 are considered significant.

## Results

The combined group (n = 280), separated according to the aetiology of amblyopia, consisted of ninety-seven anisometropic amblyopes (A, 44% Female), eighty-three strabismic amblyopes (S, 47% Female) and one-hundred combined mechanism amblyopes (C, 60% Female). The mean age for the three groups was 4.75 ± 0.93, 4.31 ± 1.03 and 4.55 ± 0.96 years, respectively. At baseline, the average VAAE was 0.56 ± 0.22 logMAR for the ATS5 participants and 0.64 ± 0.24 logMAR for the ATS13 participants.

Baseline clinical characteristics, final results (i.e. at the end of the OTA phase) and changes in the VAAE and in the interocular difference in VA (IOD-VA), in the tropia size and in stereoacuity are summarized in Table 1. The average OTA duration was 160 ± 69 days, with fifteen children taking 300 days and two cases of 378 days. The average OTA duration was shortest for the A group (151 ± 73 days), followed by the S (158 ± 63 days) and C groups (171 ± 70 days) (Table 1) but these differences are not statistically significant (Kruskal Wallis χ^2^ = 4.023; p = 0.134).

**Fig. 2:**
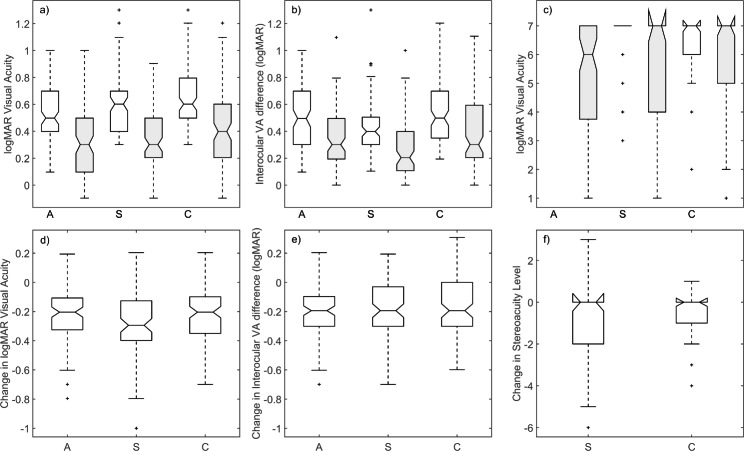
Distribution of initial (white) and final (gray) values of the outcome variables a) VAAE, b) IOD-VA and c) stereoacuity and changes in these three variables ((d)-(f)) in the three study groups (anisometropic (A), strabismic (S) and combined-mechanism (C) amblyopes). In panels d) to f), negative values correspond to improved performance following the OTA phase. The number of participants is n = 280 for a), b), d) and e); n = 235 for c) and n = 118 for f). Boxes represent the interquartile interval and whiskers cover the data at largest distance from the median that cannot be considered outliers. The narrowing of the box around the median (“notches”) define the 95% confidence interval of the median. Stereoacuity levels range from 1 (40”) to 7 (> 800”). Initial stereoacuity data for the anisometropic group were unavailable

For each of these three groups, OTA produces significant improvements in VAAE and IOD-VA (Table 1 and Fig. 2). The percentage of resolved amblyopia using PEDIG’s criterion (≤1 IOD-VA) was 22.7% (95% CI, 15.2%-31.7%) in group A, 24.1% (95% CI, 15.9%-34.1%) in group S and 13% (95% CI, 7.5%-20.6%) in group C. Overall, OTA resolved the amblyopia in 19.6% of amblyopic children (CI, 15.3%-24.6%), with no significant differences between the three groups (χ^2^ = 4.406; p = 0.110). The S and C groups showed statistically significant stereoacuity improvement (Table 1, Fig. 1), with no significant difference between the two groups (Kruskal Wallis χ^2^ = 3.011; p = 0.763). Since there were no baseline stereoacuity measures in the anisometropic amblyopes, the anisometropic group could not be included in this analysis. It is observed (Table 1) that the S group has higher tropia at baseline (both at distance and near vision) than the C group. Both groups significantly reduction with spectacles, but in the combined-mechanism amblyopia group this reduction is smaller.

**Table 1:** Summary of the sample (n = 280) extracted from PEDIG’s ATS13 and ATS5. Comparison between three groups (anisometropic, strabismic and mixed amblyopia) in baseline, final and change for: VAAE (LogMAR), interocular difference in VA (IOD-VA) (LogMAR), Tropia at Distance (Δ = prismatic diopter), Tropia at Near (Δ), stereoacuity level [1 (40”) to 7 (> 800”)] and elapsed time (days) for each group and for the whole sample, from baseline to the end of the OTA phase. Initial stereoacuity data for the anisometropic group were unavailable. The non-parametric Wilcoxon test has been used to compare baseline and end-of-OTA measures

Presumed Aetiology of Amblyopia												
	Anisometropic (A) n = 97				Strabismus (S) n = 83				Mixed mechanism (C) n = 100			
	Baseline	Final	Change	P value Wilcoxon	Baseline	Final	Change	P value Wilcoxon	Baseline	Final	Change	P value Wilcoxon
VA AE LogMar Mean ± SD Median	0.53 ± 0.22 0.50	0.29 ± 0.23 0.30	-0.24 ± 0.17 0.20	Z= -8,097 P < 0.001*	0.60 ± 0.23 0.60	0.33 ± 0.23 0.30	-0.27 ± 0.21 0.29	Z= -7,395 P < 0.001*	0.67 ± 0.23 0.60	0.44 ± 0.30 0.40	-0.23 ± 0.19 0.20	Z= -7,639 P < 0.001*
IOD LogMAR Mean ± SD Median	0.50 ± 0.23 0.49	0.32 ± 0.22 0.30	-0.18 ± 0.18 -0.19	Z= -7,285 P < 0.001*	0.46 ± 0.20 0.40	0.28 ± 0.22 0.20	-0.18 ± 0.18 -0.19	Z= -6,806 P < 0.001*	0.56 ± 0.25 0.50	0.41 ± 0.28 0.30	-0.15 ± 0.19 -0.19	Z= -6,284 P < 0.001*
Tropia Distance (∆) Mean ± SD Median Percentile (P25, P75)	-	-	-	-	10 ± 10 8 (4, 15)	4 ± 6 2 (0, 6)	6 ± 10 2 (0, 10)	Z= -5,227 P < 0.001*	7 ± 8 4 (2, 12)	4 ± 6 2 (0, 5)	3 ± 5 2 (0, 4)	Z= -3,618 P < 0.001*
Tropia Near (∆) Mean ± SD Median Percentile (P25, P75)	-	-	-	-	13 ± 10 10 (5, 20)	7 ± 7 4 (2, 12)	6 ± 1 4 (2, 10)	Z= -5,766 P < 0.001*	9 ± 8 7 (2, 14)	7 ± 7 4 (2, 10)	2 ± 5 2 (0, 5)	Z= -4,459 P < 0.001*
Stereo (levels 1 to 7) Mean ± SD	-	4.81 ± 2.02	-	-	6.49 ± 1.01	5.81 ± 1.79	-0.96 ± 0.23	Z= -3.810 P < 0.001*	6.51 ± 0.98	5.82 ± 1.69	-0.66 ± 0.15	Z= -3.899 P < 0.001*
Optical treatment period (days)		151 ± 73			158 ± 63				171 ± 70			
Global average Optical treatment for 3 groups (days)				160.3 ± 69								

**Fig. 3:**
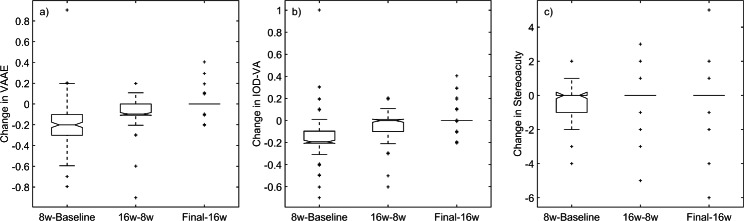
Changes between visits for VAAE (left), IOD-VA (centre) and stereoacuity (right). Not all patients had data for the intermediate visits: n = 148 for VAAE and IOD-VA. Stereoacuity is reported only for strabismic and mixed amblyopes. The y-axis in each plot shows the change in the clinical measure between the time points indicated on the x-axis. Negative values on the y-axis imply improved performance following the OTA phase. 8w: 8 weeks; 16w: 16 weeks; Final: time point when the OTA was judged to have come to an end

The time-course of the changes taking place during the OTA shows that the improvement in VAAE and IOD-VA is particularly noticeable in the first 8 weeks but the improvement continues during the subsequent 8 weeks. In stereoacuity, however, changes appear only occur between baseline and 8 weeks, except in the case of a small number of participants (outliers in Fig. 3).

### Factors associated with better outcomes of the OTA phase

Our aims were to determine the clinical factors (e.g. presence of strabismus at distance or near, size of the angle of deviation, baseline steroacuity [where available], magnitude of anisometropia, level of compliance with glasses wear) associated with better performance at the end of the OTA phase, as measured by the improvement in VAAE, IOD-VA and stereoacuity. The partial correlations between the baseline variables (VAAE, IOD-VA, stereoacuity) and the improvement in VAAE, IOD-VA and stereoacuity, did not always show similar trends for all three groups for a given variable. In what follows here, only statistically significant correlations are described. Full details of the correlational analysis (Table_A_SuppInfo.pdf) are provided in the supporting information available at Springer nature website.

The improvements in VAAE for the whole sample and the combined (C) group are greater when there is better baseline stereoacuity (Rho = 0.238; p = 0.013 and Rho = 0.357; p = 0.009, respectively) and in cases where there is better compliance (Rho= -0.231; p = 0.016 and Rho= -0.354; p = 0.009, respectively). In the S group, greater improvements in VAAE occurred in amblyopic eyes with larger refractive error (i.e. higher initial M-values, Rho= -0.294; p = 0.050).

The improvements in the IOD-VA for all three groups are greater when there is better baseline VAAE (Rho = 0.223; p = 0.015), a larger baseline IOD-VA (Rho= -0.336; p < 0.001), better baseline stereoacuity (Rho = 0.253; p = 0.008) and better compliance (R= -0.190; p = 0.049). In group A, the improvement in IOD-VA is greater for those with larger baseline IOD-VA (Rho= -0.369; p < 0.001). In group S, the IOD-VA shows greater improvement in younger children (Rho = 0.378; p = 0.010), higher baseline spherical equivalent-values (Rho= -0.357; p = 0.016) and larger baseline IOD-VA (Rho= -0.392; p = 0.008). Finally, in the C group, the improvement in IOD-VA was associated with better baseline VAAE (Rho = 0.349; p = 0.010), a larger IOD-VA (Rho= -0.472; p < 0.001), better baseline stereoacuity (Rho = 0.338; p = 0.013) and better compliance (Rho= -0.363; p = 0.008).

The improvement in stereoacuity for group S is smaller when there is larger distance tropia (Rho = 0.326; p = 0.029). In group C, the improvement in stereoacuity is larger for older children (Rho= -0.302; p = 0.028) and in those with a smaller IOD-VA at baseline (Rho = 0.321; p = 0.019).

### Stepwise linear regression modelling

The models (Tables 2, 3 and 4) show a clear trend where the most influential factor in the improvement of VAAE and IOD-VA is their respective baseline value; hence a worse baseline VAAE shows greater improvement in VAAE and the higher the baseline IOD-VA, the greater the improvement in IOD-VA. An influence of compliance is observed with worse treatment outcomes associated with the poorest level of compliance. Overall compliance was excellent, with only eight children with ‘fair’ compliance and two with ‘poor’. These patients did not share common characteristics, although all eight were strabismic. In order of importance, the next factors are the baseline spherical-equivalent refraction value and the initial anisometropia, so that larger initial spherical-equivalent refraction is related to a greater improvement in VAAE and greater anisometropia produces smaller improvements in VAAE and IOD-VA (Table 2).

Data from the S and C groups (Table 4) show that better stereoacuity is achieved with smaller baseline tropia sizes at distance, and when there is poorer stereoacuity and smaller IOD-VA values at baseline, though the effect is less strong for IOD-VA. It is also observed that aetiology influences the results in that, at the end of the OTA, the VAAE is poorer for the combined-mechanism group compared to the group with the strabismic amblyopes. In the case of IOD-VA there is greater improvement when OTA compliance is ‘excellent’.

**Table 2:** Results of stepwise, linear regression models for changes in VAAE, IOD-VA and stereoacuity for the whole sample (n = 280 cases). The importance (I) of a predictor is the residual sum of squares with the predictor removed from the model, normalized so that the importance values sum to 1. The percentage is the model’s accuracy, defined as the adjusted R^2^. C: coefficient. Significant p-value (*, p < 0.05). Cells for model terms not contributing significantly to the model’s predictive power are empty. The factors missing from the table, do not contribute to the model. The numbers between parentheses indicate the order of importance in the model for each statistically significant variable.

	ΔVAAE 10.4%			ΔIOD-VA 14.6%			ΔStereoacuity (sg/arc) 5.2%		
Model Term	C	P value	I	C	P value	I	C	P value	I
Intercept	-0.111	0.002*		-0.088	0.004*		-246.301	0.006*	
Baseline VA-AE	-0.188	< 0.001*	0.308 (1)	0.336	0.001*	0.187 (2)	318.765	0.016*	0.485 (1)
Baseline IOD-VA				-0.571	< 0.001*	0.510 (1)			
Poor vs. Excellent Compliance	0.283	0.008*	0.210 (2) 0.210 0.210	0.233	0.019*	0.094 (4)			
Fair vs. Excellent Compliance	0.085	0.191		0.047	0.442	0.094			
Good vs. Excellent Compliance	0.022	0.575		-0.012	0.756	0.094			
Baseline spherical equivalent AE	-0.018	0.006*	0.176 (3)	-0.013	0.042*	0.063 (5)			
Initial Anisometropia	0.024	0.007*	0.175 (4)	0.025	0.002*	0.145 (3)			
No Tropia vs. Tropia at Distance	-0.045	0.084	0.070				-502.335	0.110	0.209
Baseline J45 AE	0.064	0.105	0.062						

**Table 3:** Results of stepwise, linear regression models for changes in VAAE, IOD-VA for the anisometropic group (n = 97 cases). Stereoacuity data at baseline were not gathered for the anisometropic amblyopes. The importance (I) of a predictor is the residual sum of squares with the predictor removed from the model, normalized so that the importance values sum to 1. The percentage is the model’s accuracy, defined as the adjusted R^2^. C: coefficient; Significant p-value (*, p < 0.05). Cells for model terms not contributing significantly to the model’s predictive power are empty. The factors missing from the table, do not contribute to the model. The numbers between parentheses indicate the order of importance in the model for each statistically significant variable

	ΔVAAE 12.4%			ΔIOD-VA 23.4%		
Model Term	C	P value	I	C	P value	I
Intercept	-0.117	0.009*		-0.052	0.237	
Baseline VA-AE	-0.254	0.001*	0.763 (1)	0.340	0.047*	0.163 (2)
Baseline IOD-VA				-0.759	< 0.001*	0.739 (1)
Poor vs. Excellent Compliance						
Fair vs. Excellent Compliance						
Good vs. Excellent Compliance						
Baseline spherical equivalent AE						
Initial Anisometropia				0.023	0.122	0.098
No Tropia vs. Tropia at Distance						
Baseline J45 AE	0.087	0.064	0.237			

**Table 4:** Results of stepwise, linear regression models for changes in VAAE, IOD-VA and stereopsis for the strabismic (group S) and combined-mechanism (C) amblyopes (n = 117 participants, 66 out of 183 excluded for incomplete data). T The importance (I) of a predictor is the residual sum of squares with the predictor removed from the model, normalized so that the importance values sum to 1. The percentage is the model’s accuracy, defined as the adjusted R^2^. C: coefficient. Significant p-value (*, p < 0.05). Cells for model terms not contributing significantly to the model’s predictive power are empty. The factors missing from the table, do not contribute to the model. The numbers between parentheses indicate the order of importance in the model for each statistically significant variable

	ΔVA (23.1%)			ΔIOD-VA (22.7%)			ΔStereoacuity (sg/arc) (14.3%)		
Model Term	C	P value	I	C	P value	I	C	P value	I
Intercept	-0.255	< 0.001*		-0.401	< 0.001*		-115.091	0.328	
Baseline VA-AE	-0.229	0.003*	0.233 (2)	0.383	0.007*	0.127 (4)			
Baseline IOD-VA				-0.586	< 0.001*	0.287 (1)	328.207	0.014*	0.201 (3)
Baseline Stereo	< 0.001	0.011*	0.050 (5)	< 0.001	0.004*	0.146 (3)	-0.315	0.005*	0.265 (2)
Mixed vs. Strabismic	0.096	0.004*	0.220 (3)	0.089	0.005*	0.143 (5)	103.338	0.076	0.103
Poor vs. Excellent Compliance	0.457	0.009*	0.254 (1)	0.447	0.006*	0.186 (2)			
Fair vs. Excellent Compliance	0.202	0.101	0.254	0.203	0.072	0.186			
Good vs. Excellent Compliance	0.056	0.325	0.254	0.022	0.683	0.186			
Baseline spherical equivalent AE	-0.020	0.029*	0.123 (4)	-0.015	0.079	0.054	-28.800	0.061	0.115
Baseline NHT Size							9.534	0.002*	0.315 (1)
Age				0.029	0.070	0.057			

**Fig. 4:**
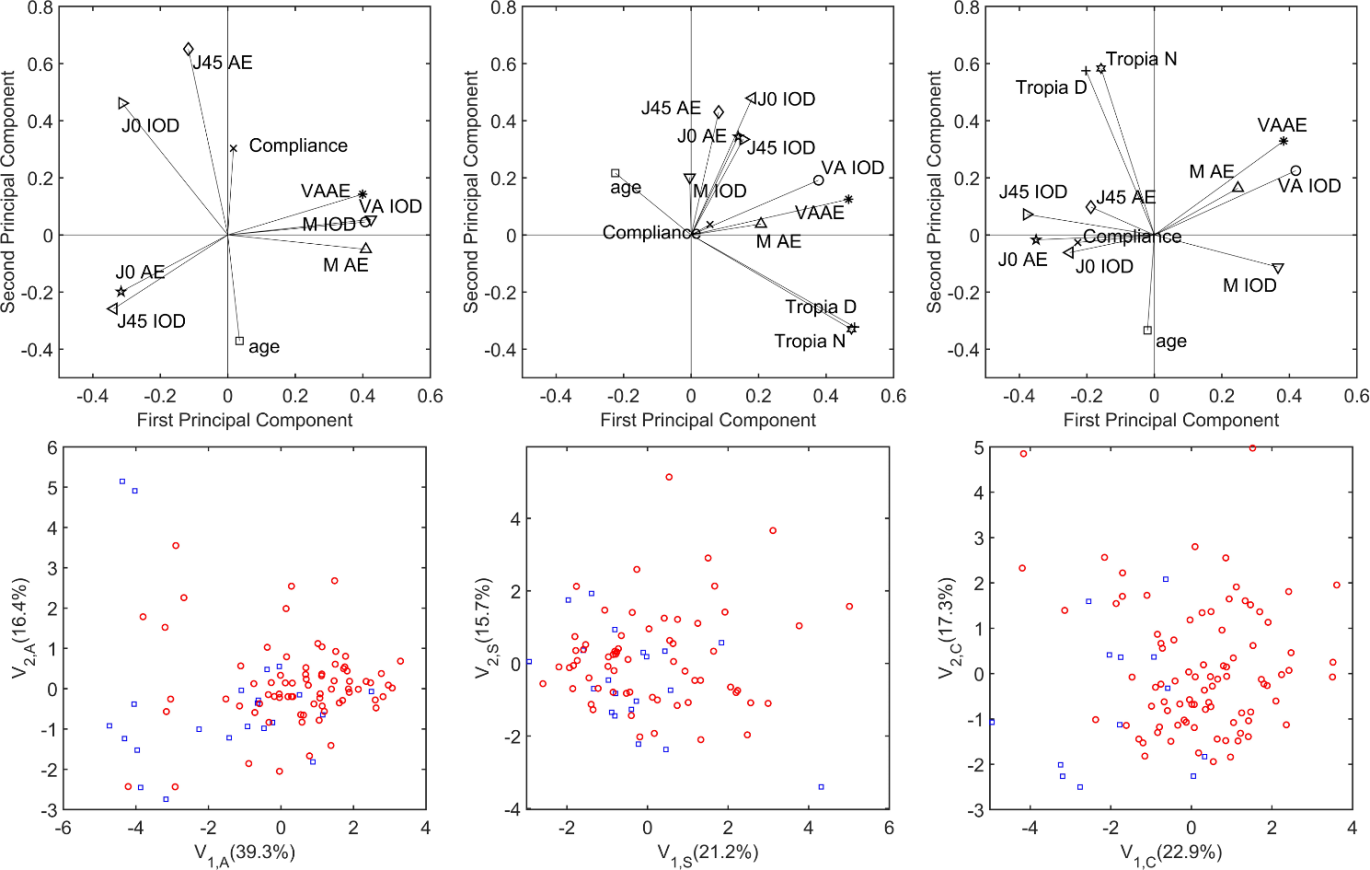
Top: First and second principal components of the baseline variables used in the PCA analysis (IOD indicates the interocular difference for the indicated variable). Bottom: Values of the first (V_1_) and second (V_2_) principal components for the anisometropic (left), strabismic (center) and combined-mechanism (right) amblyopic samples. Blue squares: resolved amblyopia; Red circles: unresolved amblyopia. The percentage of explained variance appears between brackets

The different linear models analyzed in this work fail to predict whether OTA is sufficient to resolve amblyopia. Principal component analysis [16] in the three groups (A, S & C) shows a large overlap between resolved and unresolved amblyopia in the variable space (Fig. 4), which explains the failure of the models. Using the first and second principal components, we can only predict which patients are *less likely* to resolve their amblyopia (e.g. combined mechanism amblyopia with negative first principal component scores).

## Discussion

Our analysis of the results from the two PEDIG studies confirms the effectiveness of OTA-phase in the treatment of amblyopia. This conclusion has also been reached by the authors of other studies [4–7] and by Asper et al. [10] who conducted a systematic review and meta-analysis on the topic of the optical treatment of amblyopia.

In conducting this analysis of the combined datasets from the two PEDIG studies, our aim was to identify the factors that are, or appear to be, linked to better OTA outcomes. One obvious outcome measure is the proportion of cases where the amblyopia is resolved following the OTA phase. Our primary components analysis indicates that we are not able to use an assortment of clinical factors to successfully predict the individuals in whom amblyopia will be resolved by OTA. However, this is not altogether surprising given that optical treatment by itself only leads to resolution of amblyopia in 19.6% of cases. The results of Gao et al. present similar percentages although the age of the sample is different [17]. Overall, resolution of amblyopia with optical treatment alone has been reported in 32% of patients, and the remaining patients therefore may require additional therapy [3]. The characteristics of the patients in whom amblyopia is resolved following the OTA period do not conform to a simple pattern. Those in whom OTA leads to resolved amblyopia tend to have at baseline, lower amblyopic eye spherical equivalent values, greater IOD-VA and poorer VAAE, and be without tropia or have smaller tropia sizes and have worse non-significant stereoacuity. However, we have not been able to adjust a reasonably successful model to predict whether OTA will be enough to resolve amblyopia in any given patient. After trying different classification models, our best results with anisometropic patients, for instance, were 90% patients correctly classified as “unresolved amblyopia”, but only 23% of solved amblyopia cases correctly detected.

Another approach to identifying the factors associated with better treatment outcomes following OTA is to use regression models to predict the improvement in performance arising from optical treatment, for example the improvement in VAAE. The results from this modelling exercise indicate that, again, the models perform poorly, explaining a maximum of 24% of the variance in the treatment outcome metric (Tables 2, 3 and 4). Indeed, in many of the models, the proportion of variance explained fell well below even this modest maximum (Tables 2, 3 and 4). Clearly, therefore, the large majority of the variation in treatment outcomes following OTA remains unexplained.

Leaving aside the poor predictive ability of the regression models, the clinical factors associated with treatment outcome relate to the severity of the amblyopia (as measured by the VA of the amblyopic eye or the difference in VA between the amblyopic and fellow eyes), the spherical equivalent refractive error of the amblyopic eye, the magnitude of the anisometropia (when present) and whether or not there was a level of compliance that was classed as ‘excellent’. Very little research has been conducted on the quantitative impact on treatment outcome of compliance with spectacle wear. PEDIG developed a questionnaire to assess the acceptability of amblyopia treatment and its effect on the child and family [18]. Also Papageorgiou et al. underline the importance of compliance in their review [19]. The significance of amblyopia severity upon treatment outcome has also been previously recognised in amblyopia treatment studies with a dedicated OTA-phase and it emerged as an important factor in the recent meta-analysis [10]. That larger anisometropia produces smaller improvements was already described by Chen et al. [20], in anisometropic participants.

In the anisometropic amblyopes, only two factors emerged from the modelling as being significantly related to the treatment outcome, namely the severity of the amblyopia as measured by the VA in the amblyopic eye and the magnitude of the inter-ocular VA difference. Larger improvements tend to occur (in VAAE or in the IOD-VA) in patients with poorer VA at the beginning of spectacle wear. Interestingly, in the analysis of the same anisometropic amblyopia dataset, the PEDIG group concluded that better amblyopia treatment outcomes following OTA were linked to better baseline VA and lesser amounts of anisometropia. The same finding relating to the magnitude of anisometropia emerged in our analysis when the whole data set was included in the analysis. The apparent discrepancy between our result and the PEDIG finding relating treatment outcome to severity of amblyopia at baseline is explained by the fact that PEDIG quantified the chances of resolution of amblyopia for different amblyopes with different baseline amblyopic eye acuities. In our case, we quantified the relationship between amblyopia severity and treatment outcome according to improvement in VAAE rather than according to the proportion of cases resolved by OTA.

In the strabismic amblyopes (with or without anisometropia), the factors which emerge as significantly related to treatment outcome include the severity of the amblyopia (bigger improvements in deeper levels of amblyopia) and the level of compliance with spectacle wear. The aetiology is also important with poorer outcomes in combined mechanism amblyopia compared to strabismic amblyopia without anisometropia. This is consistent with previous work [21] and may be explained by fact that there are a two amblyogenic factors in the combined mechanism form of amblyopia compare to only one in strabismic amblyopia [8]. Another, though perhaps related factor linked to treatment outcome is baseline stereoacuity, where better levels at baseline carry prognostic significance for better outcomes. Being in a position to make better predictions about the likely improvement arising from optical treatment of amblyopia would be a help to clinicians because it would help them to better advise their patients about what to expect. In particular, it would help clinicians to make realistic predictions about the timescale over which the improvements from spectacles are likely to take place, and about the likelihood that optical treatment alone will lead to a resolution of the amblyopia (i.e. without the need for further treatment methods). Unfortunately, however, our modelling indicates that accurate predictions about treatment outcomes from spectacles cannot be made for individual patients.

Our models are not restricted to visual acuity. Stereoacuity improves shortly after spectacle treatment, so binocular vision should be considered to see if amblyopia has resolved or not. The importance of considering binocular function in the treatment of amblyopia has been emphasised by Levi et al. [22].

In summary, the parameters that best predict the degree of success of the OTA-phase in amblyopic children aged from 3 to < 7 years, are the initial VAAE, the initial IOD-VA, the initial stereo acuity, compliance with spectacle wear and the amblyopic eye spherical-equivalent refractive error. Across this age range, age is not relevant to the OTA treatment outcome. While the aetiology of the amblyopia does not exert a major influence upon the treatment outcomes, this analysis shows that combined-mechanism amblyopia (anisometropia and strabismus) patients are the group with the smallest improvement in VAAE, tropia and stereoacuity and they may take longer to achieve the improvements that arise from OTA. However, despite consideration of a wide range of clinical factors and the inclusion of compliance with spectacle wear in the analysis, our models explain only a very modest proportion of the variance in treatment outcome arising from OTA. Thus, we are forced to conclude that while we know something about the factors influencing OTA treatment outcome, clinicians will not be able to make accurate predictions about the benefits of OTA in individual patients. Whether this is because the clinical factors that do predict the magnitude of the benefit from OTA have not yet been identified or because other factors may be involved (e.g. daily activities undertaken) remains to be discovered.

### Electronic supplementary material

Below is the link to the electronic supplementary material.

Supplementary Material 1

## Data Availability

are available to readers in PEDIG wesite https://public.jaeb.org/pedig.
