# Correction: Factors affecting the benefit of glasses alone in treating childhood amblyopia: an analysis of PEDIG data

**DOI:** 10.1186/s12886-023-03170-2

**Published:** 2023-10-24

**Authors:** Rosa Hernández-Andrés, María Josefa Luque, Miguel-Ángel Serrano, Andrew Scally, Brendan T Barrett

**Affiliations:** 1 Department of Optics and Optometry and Vision Science, University of Valencia, Doctor Moliner, 50, 46100 Burjassot, Spain; 2 Department of Psychobiology, University of Valencia, Avda. Blasco Ibañez, 13, 46010 Valencia, Spain; 3 School of Clinical Therapies, University College Cork, College Road, T12 K8AF Cork, Republic of Ireland; 4 School of Optometry & Vision Science, University of Bradford, Phoenix South West Building, BD7 1DP, Bradford West Yorkshire, UK

**Correction:** *BMC Ophthalmol* **23** **, 396 (2023)**

10.1186/s12886-023-03116-8

Following publication of the original article [1], it was noticed that the author name Rosa Hernández-Andrés was incorrectly written as Rosa T Hernández-Andrés.

The author group has been updated above and the original article has been corrected.

## References

[1] Hernández-Andrés R, Luque MJ, Serrano MÁ (2023). Factors affecting the benefit of glasses alone in treating childhood amblyopia: an analysis of PEDIG data. BMC Ophthalmol.

