# Supplementary material for: Factors affecting the benefit of glasses alone in treating childhood amblyopia: an analysis of PEDIG data
Source: BMC Ophthalmol. 2023 Sep 28;23:396. doi: 10.1186/s12886-023-03116-8 (PMC10540398; doi:10.1186/s12886-023-03116-8)
Supplement: Supplementary file 1 — Supplementary Material 1 [file 12886_2023_3116_MOESM1_ESM.docx]

**PARTIAL CORRELATIONS**

Table A. Partial correlations between baseline and outcome variables -change in Visual Acuity Ambliopic Eye (VAAE), change in interocular difference in VA (IOD-VA) and change in stereoacuity-. Refraction is expressed in vectorial form, with components (M, J_0_, J_45_). Correlations are computed for the whole sample (W) (n=280) and for the Anisometropic (A) (n= 97), Strabismic (S) (n=83) and Combined-mechanism (C) (n=100) groups. There is no stereoacuity and tropia data for the Anisometropic group in the original database. Significant correlations (*p*<0.05) appear in red.

| **Outcome Results** | **Group** | **Initial Age** | | **Baseline Visual Acuity AE** | | **Baseline M** | | **Baseline J_0_** | | **Baseline J_45_** | | **Baseline Tropia Distance** | | **Baseline Tropia Near** | | **Baseline Interocular Difference VA** | | **Baseline Stereoacuity** | | **Anisometropia** | | **Compliance of the Optical Treatment Alone (OTA)** | |
| --- | --- | --- | --- | --- | --- | --- | --- | --- | --- | --- | --- | --- | --- | --- | --- | --- | --- | --- | --- | --- | --- | --- | --- |
|  |  | Rho | *p* | Rho | *p* | Rho | *p* | Rho | *p* | Rho | *p* | Rho | *p* | Rho | *p* | Rho | *p* | Rho | *p* | Rho | *p* | Rho | *p* |
| **Change in VAAE** | W | -0.020 | 0.837 | -0.184 | 0.056 | -0.162 | 0.094 | 0.051 | 0.600 | 0.044 | 0.651 | -0.042 | 0.666 | 0.002 | 0.985 | 0.061 | 0.531 | 0.238 | 0.013 | 0.137 | 0.159 | -0.231 | 0.016 |
|  | A | -0.121 | 0.255 | -0.188 | 0.076 | -0.064 | 0.547 | -0.027 | 0.804 | 0.160 | 0.131 | - | - | - | - | 0.030 | 0.781 | - | - | 0.084 | 0.433 | 0.032 | 0.765 |
|  | S | 0.150 | 0.326 | -0.150 | 0.327 | -0.294 | 0.050 | 0.103 | 0.499 | 0.151 | 0.324 | -0.083 | 0.589 | 0.054 | 0.723 | -0.009 | 0.954 | 0.114 | 0.454 | 0.019 | 0.904 | -0.101 | 0.510 |
|  | C | -0.081 | 0.563 | -0.071 | 0.614 | -0.142 | 0.311 | -0.086 | 0.539 | 0.014 | 0.922 | -0.008 | 0.953 | 0.006 | 0.966 | -0.044 | 0.754 | 0.357 | 0.009 | -0.010 | 0.941 | -0.354 | 0.009 |
| **Change in**  **IOD-VA** | W | 0.130 | 0.180 | 0.223 | 0.015 | -0.146 | 0.131 | 0.042 | 0.669 | -0.003 | 0.977 | -0.043 | 0.662 | 0.016 | 0.868 | -0.336 | <0.001 | 0.253 | 0.008 | 0.175 | 0.070 | -0.190 | 0.049 |
|  | A | -0.045 | 0.671 | 0.169 | 0.111 | -0.080 | 0.451 | -0.024 | 0.824 | 0.041 | 0.700 | - | - | - | - | -0.369 | <0.001 | - | - | 0.166 | 0.119 | 0.082 | 0.440 |
|  | S | 0.378 | 0.010 | 0.286 | 0.057 | -0.357 | 0.016 | 0.144 | 0.347 | 0.248 | 0.101 | -0.108 | 0.478 | 0.126 | 0.411 | -0.392 | 0.008 | 0.178 | 0.241 | -0.002 | 0.990 | 0.009 | 0.954 |
|  | C | 0.052 | 0.710 | 0.349 | 0.010 | -0.075 | 0.595 | -0.136 | 0.331 | -0.082 | 0.557 | 0.019 | 0.892 | -0.020 | 0.885 | -0.472 | <0.001 | 0.338 | 0.013 | 0.022 | 0.873 | -0.363 | 0.008 |
| **Change in Stereo** | W | -0.159 | 0.100 | -0.019 | 0.848 | -0.170 | 0.079 | 0.027 | 0.778 | 0.034 | 0.724 | 0.163 | 0.092 | -0.066 | 0.498 | 0.111 | 0.255 | -0.124 | 0.200 | 0.145 | 0.134 | -0.092 | 0.344 |
|  | A | - | - | - | - | - | - | - | - | - | - | - | - | - | - | - | - | - | - | - | - | - | - |
|  | S | 0.007 | 0.962 | 0.248 | 0.100 | -0.187 | 0.218 | -0.033 | 0.832 | 0.117 | 0.444 | 0.326 | 0.029 | -0.199 | 0.189 | -0.226 | 0.136 | -0.282 | 0.061 | -0.023 | 0.881 | -0.086 | 0.576 |
|  | C | -0.302 | 0.028 | -0.165 | 0.237 | -0.160 | 0.253 | 0.083 | 0.554 | 0.042 | 0.765 | 0.039 | 0.784 | 0.030 | 0.831 | 0.321 | 0.019 | -0.022 | 0.876 | 0.089 | 0.525 | 0.001 | 0.996 |
